# An Algerian Soil-Living *Streptomyces alboflavus* Strain as Source of Antifungal Compounds for the Management of the Pea Pathogen *Fusarium oxysporum* f. sp. *pisi*

**DOI:** 10.3390/jof10110783

**Published:** 2024-11-12

**Authors:** Marco Masi, Dorsaf Nedjar, Moustafa Bani, Ivana Staiano, Maria Michela Salvatore, Karima Khenaka, Stefany Castaldi, Jesus Garcia Zorrilla, Anna Andolfi, Rachele Isticato, Alessio Cimmino

**Affiliations:** 1 Department of Chemical Sciences, University of Naples Federico II, Complesso Universitario Monte S. Angelo, Via Cinthia 4, 80126 Naples, Italy; mariamichela.salvatore@unina.it (M.M.S.); jesus.zorrilla@uca.es (J.G.Z.); andolfi@unina.it (A.A.); alessio.cimmino@unina.it (A.C.); 2 Laboratory of Biotechnology, Higher National School of Biotechnology Taoufik Khaznadar, Nouveau Pôle Universitaire Ali Mendjeli, BP. E66, Constantine 25100, Algeria; dorsafnedjar0@gmail.com (D.N.); khenakak@yahoo.fr (K.K.); 3 Department of Biology, University of Naples Federico II, Complesso Universitario Monte S. Angelo, Via Cinthia 4, 80126 Naples, Italy; ivana.staiano@unina.it (I.S.); stefany.castaldi@unina.it (S.C.); isticato@unina.it (R.I.); 4 Allelopathy Group, Department of Organic Chemistry, Facultad de Ciencias, Institute of Biomolecules (INBIO), University of Cadiz, C/Avenida República Saharaui, s/n, 11510 Puerto Real, Spain

**Keywords:** fusarium wilt, *Fusarium oxysporum*, *Streptomyces* sp., antimicrobial compounds, prodiginine natural products, metacycloprodigiosin

## Abstract

Fusarium wilt caused by *Fusarium oxysporum* f. sp. *pisi* (*Fop*) poses significant threats to pea cultivation worldwide. Controlling this disease is mainly achieved through the integration of various disease management procedures, among which biological control has proven to be a safe and effective approach. This study aims to extract and identify antifungal secondary metabolites from *Streptomyces alboflavus* KRO3 strain and assess their effectiveness in inhibiting the in vitro growth of *Fop*. This bacterial strain exerts in vitro antagonistic activity against *Fop*, achieving highly significant inhibition over one week. The ethyl acetate extract, obtained from its ISP2 agar medium culture, also exhibited strong antifungal activity, maintaining an inhibition rate of approximately 90% at concentrations up to 250 µg/plug compared to the control. Thus, the organic extract has been fractionated using chromatographic techniques and its bioguided purification allowed us to isolate the main bioactive compound. This latter was identified as metacycloprodigiosin using nuclear magnetic resonance (NMR) spectroscopy, electrospray ionization mass spectrometry (ESI-MS), and specific optical rotation data. Metacycloprodigiosin demonstrates dose-dependent inhibitory activity against the phytopathogen with an effective concentration of 125 µg/plug. The other secondary metabolites present in the ethyl acetate extract were also identified by gas chromatography–mass spectrometry (GC-MS) and nuclear magnetic resonance (NMR). This study highlighted the potential of *S. alboflavus* KRO3 strain and its antimicrobial compounds for the management of the pea pathogen *Fusarium oxysporum* f. sp. *pisi*.

## 1. Introduction

Fusarium wilt caused by *Fusarium oxysporum* f. sp. *pisi* (*Fop*) is an important damaging disease of field pea crops, as it has the potential to cause devastating yield losses of up to 100% in optimal conditions [1,2,3]. Its control is mainly achieved through the integration of various disease management procedures including chemical fungicides [4], resistant cultivars and breeding [5], as well as agronomic and farming practices [6,7]. However, wilt remains a major problem due to the highly variable nature of the pathogen, in addition to the adverse environmental and health consequences of used chemicals. Thus, biological management using microbial biocontrol agents may provide a sustainable, cost-effective, and environmentally friendly alternative approach to prevent fusarium wilt [2,8]. In this respect, Actinobacteria have garnered significant attention for their antifungal properties against plant pathogenic fungi [9]. These Gram-positive bacteria possess substantial biosynthetic capacity to produce a wide array of structurally diverse secondary metabolites with remarkable biological activities [10]. Notably, research indicates that approximately 70% of known antimicrobial and antifungal compounds are derived from Actinobacteria [11,12,13]. These extensive biosynthetic capabilities position them as valuable sources for the discovery and development of novel antifungal compounds for the management of plant disease, particularly fusarium wilt. Among the Actinobacteria, *Streptomyces* species are of exceptional interest as they are a potential source of antimicrobial compounds that play a crucial role in protecting plants against phytopathogen attacks [13,14,15]. In this context, the present work focuses on the chemical and biological characterization of the major bioactive metabolite produced by an Algerian soil-living *Streptomyces alboflavus* presenting significant, in vitro, antifungal activity against *F. oxysporum* f. sp. *pisi*.

## 2. Materials and Methods

### 2.1. General Experimental Procedures

Analytical and preparative thin-layer chromatography (TLC) was performed on silica gel (Kieselgel 60, F_254_, 0.25 and 0.5 mm, respectively; Merck, Darmstadt, Germany). Spots were visualized by exposure to UV radiation (254 nm) and by spraying with 10% H_2_SO_4_ in methanol (MeOH) (*v*/*v*), followed by heating at 110 °C for 10 min. Sigma-Aldrich Co. (St. Louis, MO, USA) supplied all the solvents. ^1^H and ^13^C NMR spectra were recorded in deuterated chloroform (CDCl_3_) at 400/100 MHz on Bruker (Karlsruhe, Germany) Anova Advance spectrometer and the same solvent was used as internal standard. Optical rotations were measured on a Jasco P-1010 digital polarimeter (Tokyo, Japan). HRESI-TOF mass spectra were measured on an Agilent Technologies ESI-TOF 6230DA instrument in the positive ion mode (Milan, Italy).

### 2.2. Microorganism and Culture Conditions

The bacterial isolate KRO3, exhibiting broad-spectrum antifungal activity against *Fusarium oxysporum*, was isolated from the rhizosphere of *Capsicum annuum* cultivated in the locality of Guelma (36°29′ N, 07°30′ E) in Northeastern Algeria. The dry soil sample was subjected to serial dilutions and bacterial isolation was conducted as previously described [16]. The purified strain was inoculated on ISP2 agar (Yeast extract 4 g/L, Malt extract 10 g/L, Dextrose 4 g/L, Agar 20 g/L and pH 7.2) The pure culture with 20% (*v/v*) glycerol was prepared and stored at −20 °C as spores and mycelial fragments.

After genomic DNA extraction preformed as previously described [17,18], the 16S rDNA was amplified using the universal primers F27 (5′AGAGTTTGATCCTGGCTCAG3′) and R1492 (5′TACGGCTACCTTGTTACGACTT3′) [19]. Consensus sequences were analysed using both BLAST (http://www.ncbi.nlm.nih.gov/BLAST, accessed on 15 September 2024) and EzTaxon server [20]. The bacterial isolate was identified as a member of the *Streptomyces* genus and exhibited 99.57% similarity with *Streptomyces alboflavus* NRRL B-2373^T^. The sequence was deposited in GenBank under accession number PQ328212.

### 2.3. In Vitro Antifungal Activity of the Strain

The ability of the actinobacterial strain KRO3 to inhibit the growth of the phytopathogenic *Fusarium oxysporum* f. sp. *pisi* race 2 isolate F69 [21] was examined using a direct confrontation. Two 5 mm hyphal disks from one-week-old fungal culture grown on PDA plates (Potato Dextrose Agar—Potato Dextrose: Difco, Fisher Scientific Italia, Segrate (MI), Italy; supplemented with 1.5% *w/v* of agar) were manufactured and placed at a distance of approximately 2.5 cm from the center of the Petri dish containing steak bacterial pre-culture. The tested bacterium was then streaked in the center of the same dish. After seven days of incubation at 28 °C, fungi mycelial growth was measured. The bacterial antagonistic capacity was assessed by calculating the percentage of inhibition of mycelial growth compared to the control, according to the formula reported by Zdorovenko et al., 2021 [22]. Petri dishes used as controls contained only two mycelial disks of the fungal strains. The experience was repeated thrice.

### 2.4. Bacterial Growth and Extraction of Bioactive Compounds

Plates of ISP2 agar medium, inoculated by pure culture of KRO3 strain, were incubated seven days at 30 °C. Culture medium was cut into small pieces and macerated in ethyl acetate overnight at room temperature as described by Leulmi et al. (2019) [23]. The extraction was repeated twice, and the combined organic extracts (TOE_KRO3_) were dried with Na_2_SO_4_ and then evaporated under reduced pressure.

### 2.5. Antifungal Activity of the Organic Extract, Chromatographic Fractions (FA-FE), and Metacycloprodigiosin

Antifungal activities of TOE_KRO3_, chromatographic fractions (FA-FE), and pure metacycloprodigiosin were tested against the phytopathogenic fungus *Fop*. The Minimal Inhibitory Concentration (MIC) of TOE_KRO3_ was evaluated as previously reported [24]. Briefly, a weighted amount of TOE _KRO3_ was dissolved in MeOH. This solution was serially diluted to obtain solutions at different concentrations of TOE _KRO3_. Each solution (10 μL) was deposited on the center of a PDA plate. A 5 mm fungal disc of *Fop* taken from a one-week-old plate and deposited on the center of the Petri dish amended with the TOE_KRO3_ at concentrations ranging from 62.5 to 1000 µg/plug. Before depositing the fungus disc, the solvent was allowed to evaporate in a laminar flow cabinet, and the plates were incubated at 25 °C for 7 days. MeOH was used as a negative control. Fungal growth was evaluated on each plate and MIC was reported by calculating of growth percentage inhibition as previously reported [22], using the following formula:

$$Growth\;inhibition\;\left(\%\right)=\frac{\left(R_{c}-R_{i}\right)}{R_{c}}\;\cdot \;100$$

where *R_c_* is the radial growth of the test pathogen in the control plates (mm), and *R_i_* is the radial growth of the test pathogen in the test plates (mm). The experiment was performed in triplicate.

The same protocol was used also to test the antifungal activities of the chromatographic fractions (FA-FE) at a concentration of 250 µg/plug and to determine the MIC of pure metacycloprodigiosin at concentrations ranging from 15.625 to 200 µg/plug.

### 2.6. Isolation of Bacterial Metabolites

A total of 100 mg of the bacterial organic extract was purified by column chromatography (CC) using as eluent CHCl_3_/*i*-PrOH (95:5, *v/v*), yielding five groups (FA–FE) of homogeneous fractions of 18.7 (FA), 35.6 (FB), 7.4 (FC), 28.3 (FD), and 6.5 (FE) mg, respectively. The residue of FA, showing antifungal activity against *Fop*, was further purified by two steps on TLC eluted with CHCl_3_/*i*-PrOH (98:2, *v/v*) and petroleum–ether/acetone (8:2) yielding a pure amorphous solid identified as metacycloprodigiosin (**1**, 8.6 mg).

*Metacycloprodigiosin* (**1**): red amorphous solid; [α]^25^_D_ -2360 (*c* 0.1, CHCl_3_) (ref. [25] [α]^20^_D_ -2370); ESI-MS (+): *m*/*z* 392 [M + H]^+^; ^1^H NMR (400 MHz, CDCl_3_) 12.78 (bs, 1H, NH), 12.65 (bs, 1H, NH), 12.59 (bs, 1H, NH), 7.23 (m, 1H, H-1), 7.06 (s, 1H, H-9), 6.92 (m, 1H, H-3), 6.35 (m, 1H, H-2), 6.27 (d, 1H, *J* = 1.6 Hz, H-12), 6.10 (s, 1H, H-6), 4.03 (s, 3H, Me-25), 3.22 (m, 1H, H-14A), 2.76 (m, 1H, H-14B), 2.54 (m, 1H, H-22), 1.82–0.24 (m, 16H, H_2_-15-21 and H_2_-23), 0.89 (t, 3H, *J* = 6.2 Hz, Me-24); ^13^C NMR (100 MHz, CDCl_3_) 165.5 (C-7), 154.5 (C-13), 150.4 (C-11), 147.4 (C-5), 126.8 (C-1), 126.0 (C-10), 122.3 (C-4), 120.6 (C-8), 116.8 (C-3), 113.3 (C-9), 112.4 (C-12), 111.6 (C-2), 92.7 (C-6), 58.7 (C-25), 39.6 (C-22), 29.9 (C-23), 29.1 (C-14), 34.4, 27.3, 26.8, 26.6, 25.6, 24.5, 22.7 (C-15-21), 12.6 (C-24).

### 2.7. GC-MS Analysis of Fractions FB-FE

GC-MS data were acquired on chromatographic fractions (FB-FE) after trimethylsilylation with *N*,*O*-bis(trimethylsilyl)trifluoroacetamide (BSTFA) (Fluka, Buchs, Switzerland) as previously reported [26]. GC-MS measurements were performed with an Agilent 6850 GC (Milan, Italy), equipped with an HP-5MS capillary column (5% phenyl methyl poly siloxane stationary phase), coupled to an Agilent 5973 Inert MS detector operated in the full scan mode (*m*/*z* 29–550) at a frequency of 3.9 Hz and with the EI ion source and quadrupole mass filter temperatures kept, respectively, at 200 °C and 250 °C. Helium was used as carrier gas at a flow rate of 1 mL/min. The injector temperature was 250 °C and the temperature ramp raised the column temperature from 70 °C to 280 °C: 70 °C for 1 min; 10 °C/min until reaching 170 °C; and 30 °C/min until reaching 280 °C. Then, it was held at 280 °C for 5 min. The solvent delay was 4 min. Compounds were identified by comparing their EI mass spectra at 70 eV with spectra present in the NIST 20 mass spectral library using NIST MS Search 2.4 (NIST 20, https://www.nist.gov/srd/nist-standard-reference-database-1a accessed on 20 October 2024). In addition, the identification was supported by the Kovats retention index (RI) calculated for each analyte by the Kovats equation, using the standard *n*-alkane mixture in the range C7-C40 (Sigma-Aldrich, Saint Louis, MO, USA).

### 2.8. Statistical Analysis

All the statistical analyses were performed using Minitab^®^ 20.4 software. Data were expressed as mean ± SEM. Differences among groups were compared by One-way ANOVA followed by a multiple comparison test whenever the *ANOVA* test was statistically significant at *p* < 0.05.

## 3. Results

### 3.1. The Strain KRO3

The physiological characteristics of strain KRO3 are summarized in Table 1. This filamentous, Gram-positive bacterium exhibits notable metabolic versatility, efficiently utilizing a broad spectrum of carbon sources for both growth and energy production. In addition, it can metabolize a variety of amino acids as nitrogen sources. Strain KRO3 has demonstrated tolerance to NaCl concentrations of up to 6% and possesses the ability to degrade gelatin and casein. The partial 16S rDNA gene sequence analysis demonstrated that the strain KRO3 was most likely *S. alboflavus* (Accession No. PQ328212).

### 3.2. In Vitro Antifungal Activity of the Strain

**Figure 1:**
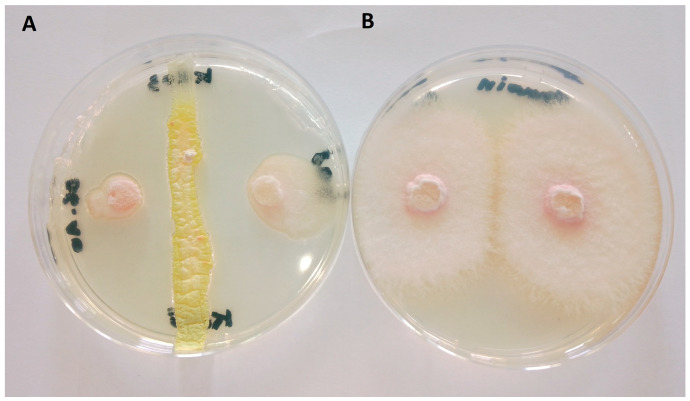
In vitro antifungal assay of the strain. (**A**) Growth inhibition of *F. oxysporum* f. sp. *pisi* by Streptomyces alboflavus KRO3; (**B**) *F. oxysporum* f. sp. *pisi* on Potato Dextrose Agar (PDA) as control.

The ability of *Streptomyces alboflavus* KRO3 to inhibit the growth of *Fop* was assessed through one-week direct confrontation test on PDA (Potato Dextrose Agar). Results revealed a significant inhibition rate of 91.00 ± 1.73 compared to the untreated control (Figure 1).

### 3.3. Antifungal Activity of the Organic Extract

**Figure 2:**
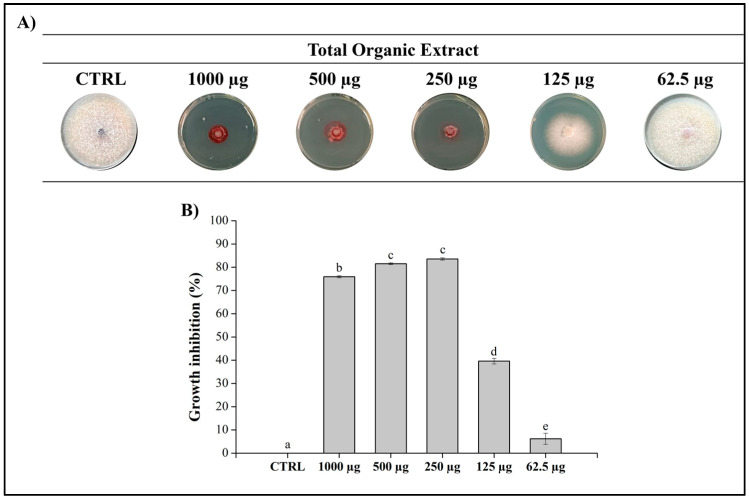
Antifungal assay of the organic extract. (**A**) The TOE_KRO3_ of *Streptomyces alboflavus* KRO3 was tested at 1000, 500, 250, 125, and 62.5 μg/plug against *Fop* grown on PDA plates for 7 days at 25 °C. MeOH was used as negative control; (**B**) graphical representation of the inhibition of the fungal growth of *Fop* by TOE_KRO3_. Data are presented as means ± S.E.M. (n = 3 replication for each concentration) compared to control *Fop* grown only with MeOH. One-way ANOVA test was performed to compare the groups of data; values that do not share a letter are statistically different (*p* < 0.05).

The bacterium isolate was cultured on ISP2 agar plates and extracted with EtOAc. The resulting organic extract (TOE_KRO3_) was analyzed for its antifungal activity against *Fop* at concentrations ranging from 100 µg/µL (1 mg/plug) to 6.25 µg/µL (62.5 µg/plug) with MeOH serving as the negative control (Figure 2). As reported in Figure 2, the *S. alboflavus* KRO3 extract exhibited strong antifungal activity, maintaining an inhibition rate of approximately 90% at concentrations up to 250 µg/plug compared to the control, indicating that the bacterial metabolites are effective against the fungus.

### 3.4. Fractionation of the Organic Extract and Evaluation of the Antifungal Activity

**Figure 3:**
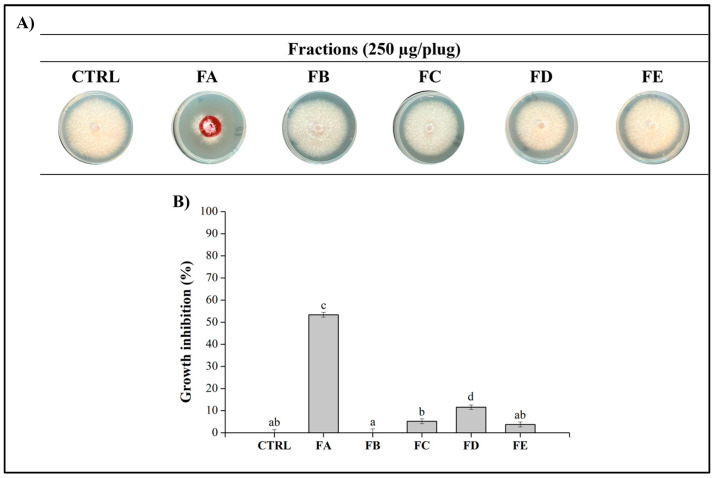
Antifungal assay of the fractions obtained from the organic extract. (**A**) The fractions of TOE_KRO3_ (from FA to FE) were tested at a concentration of 250 μg/plug against *Fop* grown on PDA plates for 7 days at 25 °C. MeOH was used as negative control. (**B**) Graphical representation of the inhibition of the fractions. Data are presented as means ± S.E.M. (n = 3 replication for each concentration) compared to control *Fop* grown only with MeOH. One-way ANOVA test was performed to compare the groups of data; values that do not share a letter are statistically different (*p* < 0.05).

Given the notable antifungal activity of TOE_KRO3_ at a concentration of 250 µg/plug, the active extract was subjected to column chromatography to isolate and identify the specific compounds responsible for this potent activity. This fractionation process yielded five distinct fractions, labeled FA through FE. To assess their efficacy against *Fop*, each fraction was tested at the same concentration of 250 µg/plug. The results illustrated in Figure 3 highlight the differential antifungal activities exhibited by these fractions. The fractions FB, FC, FD, FE showed no antagonistic activity while FA was the only fraction exhibiting significant antifungal activity, achieving a 53% inhibition in fungal growth. This substantial inhibitory effect highlights FA as the primary fraction contributing to the antifungal potency of the original extract, TOE_KRO3_.

### 3.5. Purification of Fraction FA

**Figure 4:**
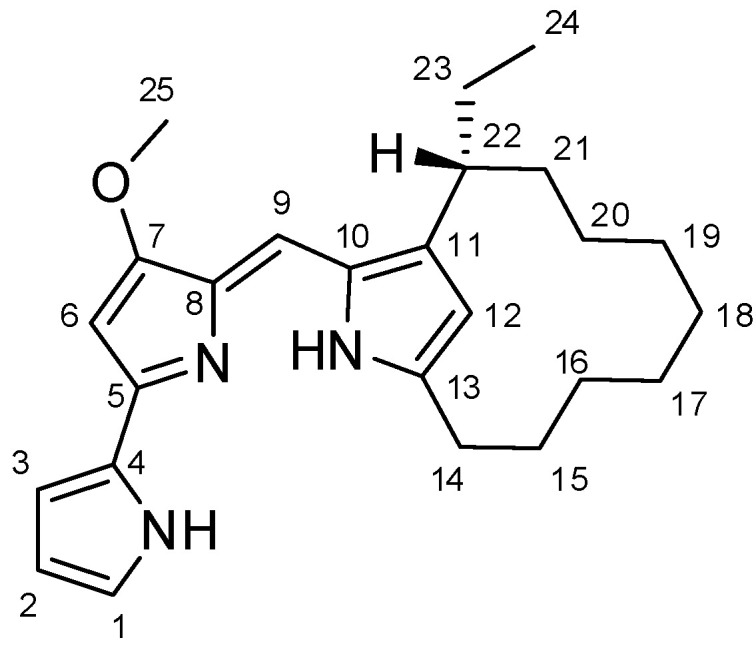
Chemical structure of metacycloprodigiosin (**1**).

Fraction A was further purified by TLC yielding a pure compound which was identified as metacycloprodigiosin (**1**, Figure 4) comparing its spectroscopic (^1^H and ^13^C NMR), spectrometric (ESI-MS), and physical (specific optical rotation) data with those reported in the literature [25,27].

In particular, its ^1^H and ^13^C NMR spectra (Figures S1 and S2) showed the typical signals patterns of the tripyrrole pigments belonging to the prodiginine family of bacterial alkaloids [28]. Furthermore, the same spectra are in agreement with the data previously reported by Kimata et al. (2017) [27] for metacycloprodigiosin (**1**). Its identification was confirmed using the data obtained from the ESI-MS spectrum recorded in the positive mode (Figure S3) that showed the protonated pseudomolecular ion [M+H]^+^ peak at *m/z* 392. Finally, its configuration was confirmed by comparing the specific optical rotation value with that reported in the literature [25].

### 3.6. Antifungal Activity of Metacycloprodigiosin

**Figure 5:**
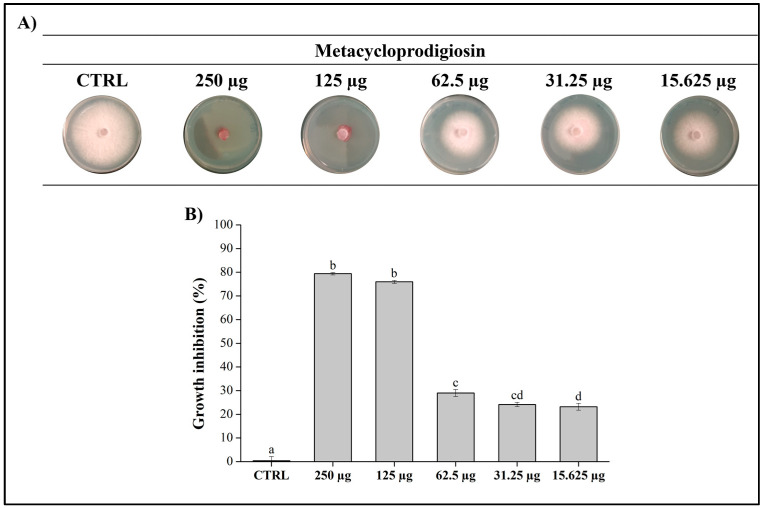
Antifungal assay of metacycloprodigiosin (**1**). (**A**) The metacycloprodigiosin was tested at 250, 125, 62.5, 31.25, and 15.625 μg/plug against *Fop* grown on PDA plates for 7 days at 25 °C. MeOH was used as negative control; (**B**) graphical representation of the inhibition of metacycloprodigiosin. Data are presented as means ± S.E.M. (n = 3 replication for each concentration) compared to control *Fop* grown only with MeOH. One-way ANOVA test was performed to compare the groups of data; values that do not share a letter are statistically different (*p* < 0.05).

To further confirm the responsibility of metacycloprodigiosin for the antifungal activity, we tested the pure compound on *Fop* at different concentrations to determine the MIC. By systematically varying the concentration of metacycloprodigiosin, we aimed to pinpoint the lowest concentration at which this compound effectively inhibits the growth of the fungus. Results reported in Figure 5 demonstrated a clear dose-dependent response, indicating that as the concentration of metacycloprodigiosin increased, the inhibition of fungal growth also increased correspondingly. At lower concentrations (from 62.5 µg/plug down), the antifungal activity was moderate (less than 30% of *Fop* growth inhibition), but as the concentration approached the MIC (125 µg/plug), a significant reduction in fungal growth was observed. This sharp decline in growth near the MIC confirms the potent antifungal nature of metacycloprodigiosin. As reported in Figure 5, purified metacycloprodigiosin exhibited a significantly stronger antifungal effect than fraction FA, from which it was obtained, with an inhibition percentage of about 80% compared to 53% inhibition observed in fraction FA.

### 3.7. Identification of Metabolites in Fractions FB-FE

The four chromatographic fractions FB-FE were analyzed via GC-MS after trimethylsilylation (see Section 2), and the detected compounds are listed in Table 2. Several fatty acids and their esters were identified in fractions FB and FD, while 4-nitrobenzamide and furandimethanol were detected in fractions FC and FE, respectively (Figures S4 and S5). The identification of the latter compounds was further supported by NMR spectroscopy. In fact, the ^1^H NMR spectra of fractions FC and FE, recorded in CDCl_3,_ showed the presence of 4-nitrobenzamide and furandimethanol signals, respectively (Figures S6 and S7) [29,30].

## 4. Discussion

The *Streptomyces alboflavus* KRO3 strain was characterized and found to exhibit very strong in vitro antifungal activity against *Fop* (Figure 1). The observed inhibition zone might suggest the presence of diffusible inhibitory substances in the medium that affected the growth of the pathogen (*Fop*). Previous studies described antagonistic activities of other strains of *Streptomyces* sp. against other formae speciales (ff.spp.) of *Fusarium oxysporum*, such as f. sp. *cubense* [13,31], f. sp. *lycopersici* [32,33], and f. sp. *ciceris* [34].

KRO3 was, thus, cultured in ISP2 agar medium to extract and characterize its bioactive secondary metabolites. The organic ethyl acetate extract of the sample showed an important antagonistic activity. However, the antifungal activity of the organic extract decreased slightly compared to that of the bacterium, suggesting that the bacterium’s superior efficacy may result from various factors, such as the instability of certain bacterial compounds outside of the cellular environment, the loss of synergy due to selective extraction and solubility issues, the lack of physical interaction between molecules, or the absence of an induced response in the presence of fungi and the live bacteria. The ethyl acetate extract was subsequently fractionated using silica gel column chromatography to isolate and purify its bioactive compounds. Among the five fractions obtained from the organic extract, only the FA fraction showed significant antifungal activity, with a 53% inhibition in fungal growth, highlighting that FA is the main fraction contributing to the antifungal activity of the original extract, TOEK_RO3_. Purification of bioactive fraction FA by chromatographic techniques led to the isolation of a pure compounds which was identified as metacycloprodigiosin (**1**) [35].

GC-MS analysis of the further chromatographic fractions (FB-FE) led to the identification of fatty acids and monoglycerides in fractions FB and FD. Considering that fatty acids are ubiquitous molecules that are normally found bound to other compounds such as glycerol [36], it is not surprising to detect them in our samples. These findings can be further explained by the fact that fatty acids are essential components of the streptomycetes membrane with a role in bacterial survival and adaptation to different environmental conditions [37]. In the FC and FE fractions, 4-nitrobenzamide and furandimethanol were identified, confirming the ability of these bacteria to produce chemically diverse metabolites [38]. However, as said above, these four chromatographic fractions turned out to be very poorly active in inhibiting *Fop* (Figure 3).

Metacycloprodigiosin, isolated from fraction FA, is a derivative of prodigiosin which is an important natural red pigment, produced as a secondary metabolite by microorganisms [28]. Structurally, these compounds are distinguished by its unique structure consisting of three pyrrole rings and a pyrrolyl-dipyrrolyl-methene backbone, with a methoxy group at the C-4 position [39]. Other notable members of the prodiginine family include undecylprodigiosin, nonylprodigiosin, and roseophilin [28]. Prodiginine was originally isolated from the bacterium *Serratia marcescens*, but it can be secreted by many other bacteria, such as *Hahella chejuensis*, *Serratia nematodiphila*, *Streptomyces coelicolor*, *Serratia rubidaea*, and *Streptomyces grisiovirides* [40]. Metacycloprodigiosin (**1**) was first isolated from *Streptomyces longisporus ruber* by Wasserman et al. (1966) [41], together to undecylprodigiosin but the structure was elucidated only some years later [35] and it was confirmed by total synthesis of a racemic samples [25]. Clift and Thompson (2009) [42] realized the enantioselective synthesis and the absolute configuration of the natural compound was shown to be (*R*) by comparison of its electronic circular dichroism spectrum with the synthetic one [43]. Recently, Peixoto et al. (2018) [44] suggested that (*R*)-metacycloprodigiosin can exist in three different tautomeric forms and the addition of HCl reduces this structural diversity to *syn*-(*R*)-metacycloprodigiosin-HCl and *anti*-(*R*)-metacycloprodigiosin-HCl each with hydrogens at C-9′ and C-12 in *syn* or *anti* orientation [44].

The prodiginine group of compounds have become a new research area for scientists; it has attracted increasing interest due to its remarkable biological activities and diverse applications. This promising biomolecule is used in various sectors, including the food, cosmetic, textile, and pharmaceutical industries. Its properties include antimicrobial, immunosuppressive, antimalarial, antineoplastic, and anticancer effects [45,46]. Recent studies have rekindled interest in prodigiosin because of its reported profound biological activities [45]. The antifungal activity of our pure compound was evaluated against *Fop* at various concentrations to determine the MIC. The molecule showed a dose-dependent response with MIC (125 µg/plug) resulting in a significant reduction in fungal growth. This sharp decline in growth near the MIC confirms the potent antifungal nature of metacycloprodigiosin. In agricultural research, the class of prodiginines has been shown to exhibit significant activity against various plant pathogens. It was reported that prodigiosin can act against gray mold, through spore germination inhibition in *Botrytis cinerea* [47]. The purified red pigment of *Serratia marcescens* was found to be effective against plant parasitic nematodes *Radopholus similis* and *Meloidogyne javanica* [48]. Habash et al. (2020) [49] reported inhibition in the plant pathogenic fungi *Phoma lingam* and *Sclerotinia sclerotiorum*. Prodigiosin has also been observed to have a toxic effect on certain fungal species, such as *Batrachochytrium dendrobatidis*, *B. salamandrivorans*, *Pythium myriotylum*, *Rhizoctonia solani*, *Sclerotium rolfsii*, *Phytophthora infestans*, *Fusarium oxysporum*, and *Colletotrichum nymphaeae* [50].

This study investigates, for the first time, the antifungal effect of metacycloprodigiosin produced by *Streptomyces alboflavus*. The stark contrast in the efficacy between the pure metacycloprodigiosin and the FA fraction highlights the potential benefits of using the purified compound in antifungal applications. However, the difference between the 80% inhibition observed by TOE_KRO3_ and the 53% inhibition by FA alone suggests that the higher efficacy of the total extract is likely due to synergistic interactions among its various molecules. Synergy among the compounds in TOE_KRO3_ likely plays a critical role in its enhanced antifungal activity. When the inhibition percentages of all individual fractions are combined, they only total approximately 90%, which matches the inhibition percentage of the total extract, suggesting additive effects of all fractions in inhibitory activity. This indicates that while FA is highly effective on its own, the presence of other fractions contributes additional antifungal effects, enhancing the overall potency of TOE_KRO3_ beyond the sum of its parts. Thus, further purification and characterization of high amount of the TOE_KRO3_ extract could lead to the discovery of additional active compounds produced with very low yield and a better understanding of the synergistic or antagonistic interactions within the extract. On the other hand, the findings establish a foundation for the future development of metacycloprodigiosin as a plant protection agent. To achieve this, it is essential to further optimize the culture conditions for metacycloprodigiosin production to obtain higher yields.

## Figures and Tables

**Table 1:** Physiological characteristics of bacterial isolate KRO3 ^a^.

Carbon Source Utilization	Nitrogen Source Utilization	Growth in the Presence of. . .	Degradation Activity
Arabinose +	Histidine +	pH 4 −	Gelatin +
Fructose +	Arginine +	pH 7 +	Casein +
Galactose +	Tyrosine +	pH 9 +	Starch −
Inositol +	Alanine +	NaCl 6% *w*/*v* +	Cellulose −
Mannitol +	Glycine +	NaCl 8% *w*/*v* +	
Glucose +	Methionine +	NaCl 8% *w*/*v* +	
Lactose +	Valine +	Phenol 0.1% *w*/*v* +	
Rhamnose −	Leucine +	Penicillin 10 UI +	
Maltose +	Tryptophan +	Growth at 45 °C +	
Sucrose +	Asparagine +	Nitrate reduction +	
Sorbitol +	Proline +		
Xylitol +			
Mannose +			
Xylose −			

^a^ Plus sign (+) indicates a positive reaction; minus sign (−) indicates a negative reaction.

**Table 2:** Compounds identified in chromatographic fractions FB-FE obtained from organic extract of ***S.****alboflavus.* RI represents Kovats retention index and TMS is the trimethylsilyl function, (CH_3_)_3_Si.

Compound	RT (min)	RI	Peak Area%
**Fraction FB**			
Myristic acid, TMS	12.59	1814	6.88
Pentadecanoic acid, TMS	13.04	1915	45.46
Palmitelaidic acid, TMS	13.36	1994	2.65
Palmitic acid, TMS	13.42	2011	23.01
Margaric acid, TMS	13.81	2118	13.03
10-Heptadecenoic acid, TMS	13.87	2137	8.69
Oleic acid, TMS	14.07	2192	0.28
**Fraction FC**			
4-Nitrobenzamide, TMS	12.82	1864	-
**Fraction FD**			
1-Monomyrisitin, 2TMS	14.72	2362	1.14
2-Monopalmitin, 2TMS	14.98	2423	0.21
Pentadecanoic acid, glycerine-(1)-monoester, 2TMS	15.16	2461	25.88
1-Monopalmitin, 2TMS	15.66	2559	36.28
Heptadecanoic acid, glycerine-(1)-monoester, 2TMS	16.25	2652	29.86
1-Monooleoylglycerol, 2TMS	16.90	2746	6.64
**Fraction FE**			
Furandimethanol (2TMS)	9.54	1427	-

## Data Availability

Data is contained within the article or Supplementary Materials.

## Supplementary Materials

The following supporting information can be downloaded at: https://www.mdpi.com/article/10.3390/jof10110783/s1, Figure S1. ^1^H NMR spectrum of metacycloprodigiosin (1) (CHCl_3_, 400 MHz). Figure S2. ^13^C NMR spectrum of metacycloprodigiosin (**1**) (CHCl_3_, 100 MHz), Figure S3. ESI- MS spectrum of metacycloprodigiosin (**1**), recorded in positive mode. Figure S4. EI mass spectrum at 70 eV of 4-nitrobenzamide, TMS (RI = 1864). TMS = trimethylsilyl group. Figure S5. EI mass spectrum at 70 eV of furandimethanol, 2TMS (RI = 1427). TMS = trimethylsilyl group. Figure S6. ^1^H NMR spectrum of FC (CHCl_3_, 400 MHz). Figure S7. ^1^H NMR spectrum of FE (CHCl_3_, 400 MHz).
