# Supplementary material for: An Algerian Soil-Living Streptomyces alboflavus Strain as Source of Antifungal Compounds for the Management of the Pea Pathogen Fusarium oxysporum f. sp. pisi
Source: J Fungi (Basel). 2024 Nov 12;10(11):783. doi: 10.3390/jof10110783 (PMC11596007; doi:10.3390/jof10110783)
Supplement: Supplementary file 1 [file jof-10-00783-s001.zip › jof-3307314-supplementary.pdf]

SUPPORTING INFORMATION

Article

An Algerian Soil-Living *Streptomyces alboflavus* Strain as Source of Antifungal Compounds for the Management of the Pea Pathogen *Fusarium oxysporum* f. sp. *pisi*

Marco Masi [1,*,†], Dorsaf Nedjar [2,†], Moustafa Bani [2,*], Ivana Staiano [3], Maria Michela Salvatore [1], Karima Khenaka [2], Stefany Castaldi [3], Jesus Garcia Zorrilla [1,4], Anna Andolfi [1], Rachele Isticato [3] and Alessio Cimmino [1]

[1] Department of Chemical Sciences, University of Naples Federico II, Complesso Universitario Monte S. Angelo, Via Cinthia 4, 80126 Naples, Italy; mariamichela.salvatore@unina.it (M.M.S.); jesus.zorrilla@uca.es (J.G.Z.); andolfi@unina.it (A.A.); alessio.cimmino@unina.it (A.C.)
[2] Laboratory of Biotechnology, Higher National School of Biotechnology Taoufik Khaznadar, Nouveau Pôle Universitaire Ali Mendjeli, BP. E66, Constantine 25100, Algeria; dorsafnedjar0@gmail.com (D.N.); khenakak@yahoo.fr (K.K.)
[3] Department of Biology, University of Naples Federico II, Complesso Universitario Monte S. Angelo, Via Cinthia 4, 80126 Naples, Italy; ivana.staiano@unina.it (I.S.); stefany.castaldi@unina.it (S.C.); isticato@unina.it (R.I.)
[4] Allelopathy Group, Department of Organic Chemistry, Facultad de Ciencias, Institute of Biomolecules (INBIO), University of Cadiz, C/Avenida República Saharaui, s/n, 11510 Puerto Real, Spain
* Correspondence: marco.masi@unina.it (M.M.); m.bani@ensbiotech.edu.dz (M.B.)
† Authors contributed the same way to the work.

Supporting information list

Figure S1. ^{1}H NMR spectrum of metacycloprodigiosin (**1**) ($CHCl_3$, 400 MHz).

Figure S2. ^{13}C NMR spectrum of metacycloprodigiosin (**1**) ($CHCl_3$, 100 MHz).

Figure S3. ESI-MS spectrum of metacycloprodigiosin (**1**), recorded in positive mode.

Figure S4. EI mass spectrum at 70 eV of 4-nitrobenzamide, TMS (RI = 1864). TMS = trimethylsilyl group.

Figure S5. EI mass spectrum at 70 eV of furandimethanol, 2TMS (RI = 1427). TMS = trimethylsilyl group.

Figure S6. ^{1}H NMR spectrum of FC ($CHCl_3$, 400 MHz).

Figure S7. ^{1}H NMR spectrum of FE ($CHCl_3$, 400 MHz).

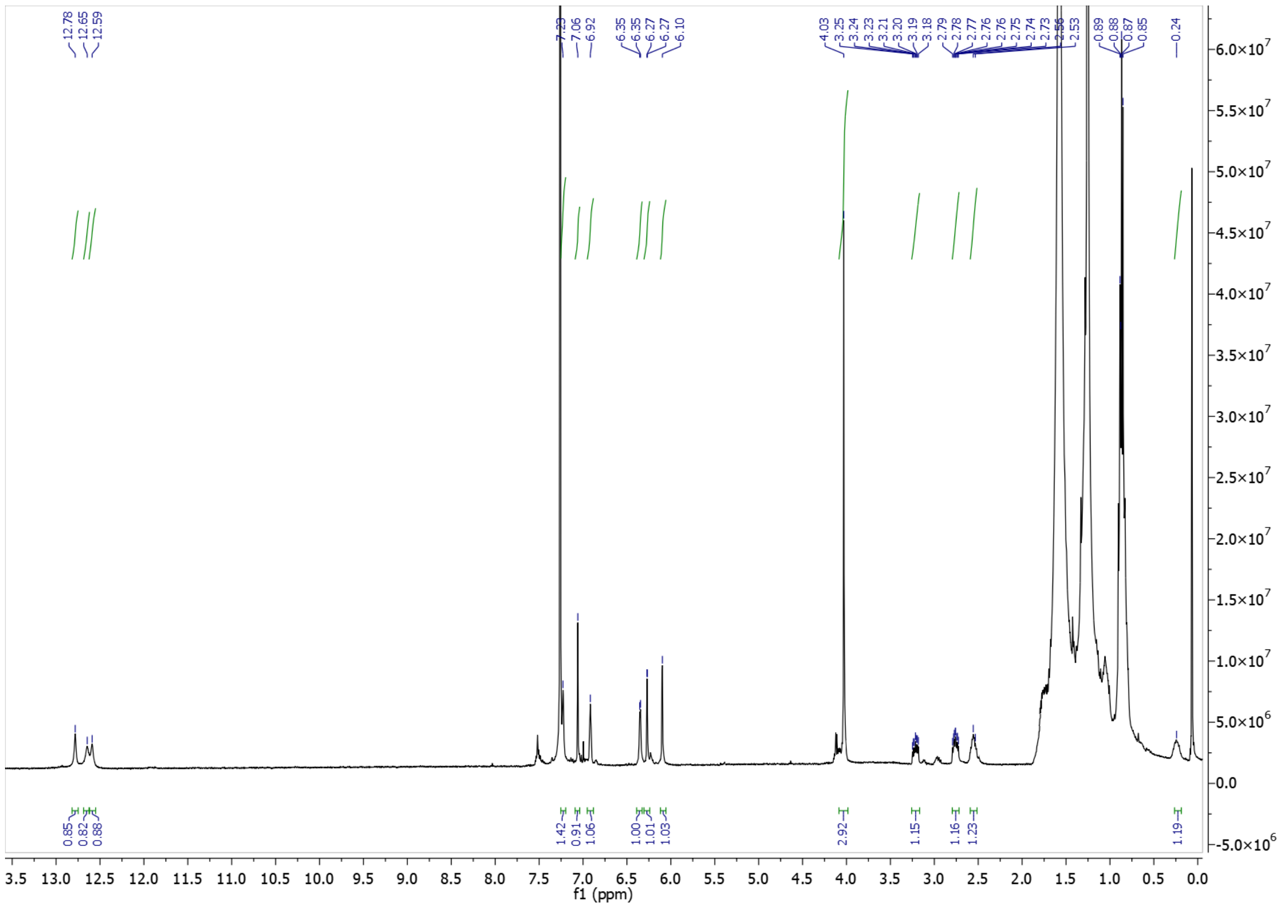

Figure S1. ^{1}H NMR spectrum of metacycloprodigiosin (**1**) ($CHCl_3$, 400 MHz).

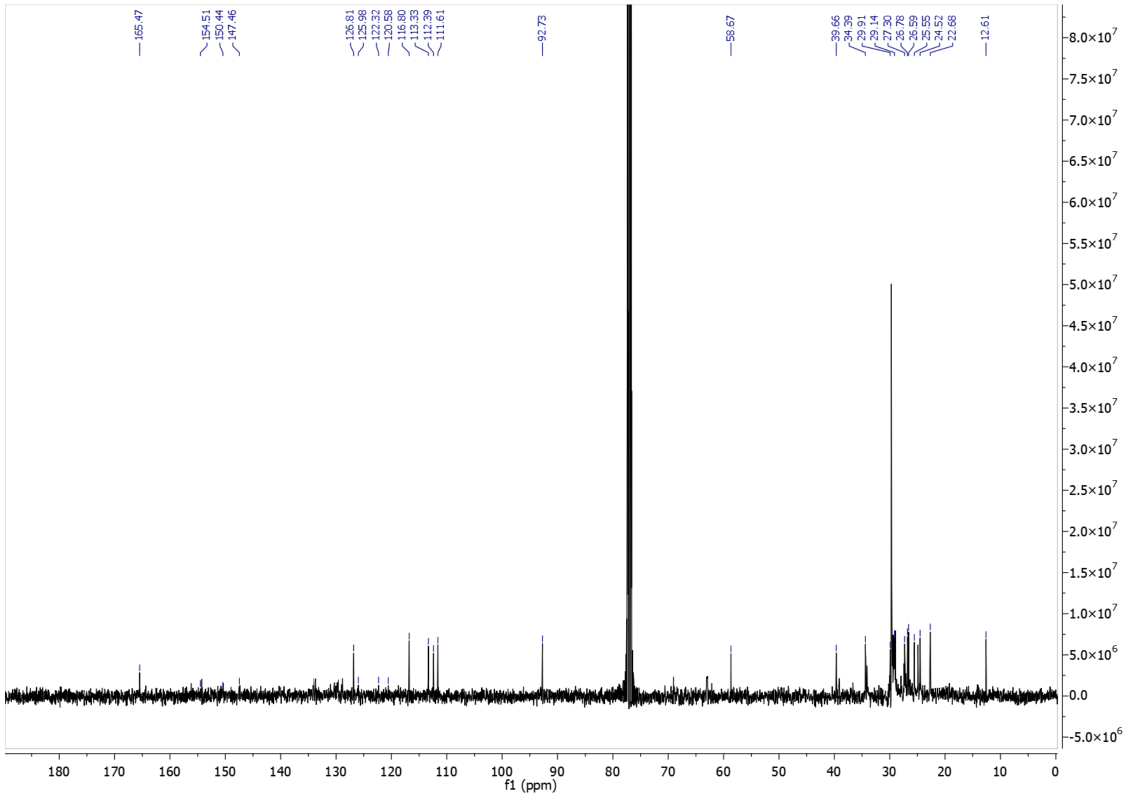

Figure S2. ^{13}C NMR spectrum of metacycloprodigiosin (**1**) ($CHCl_3$, 100 MHz).

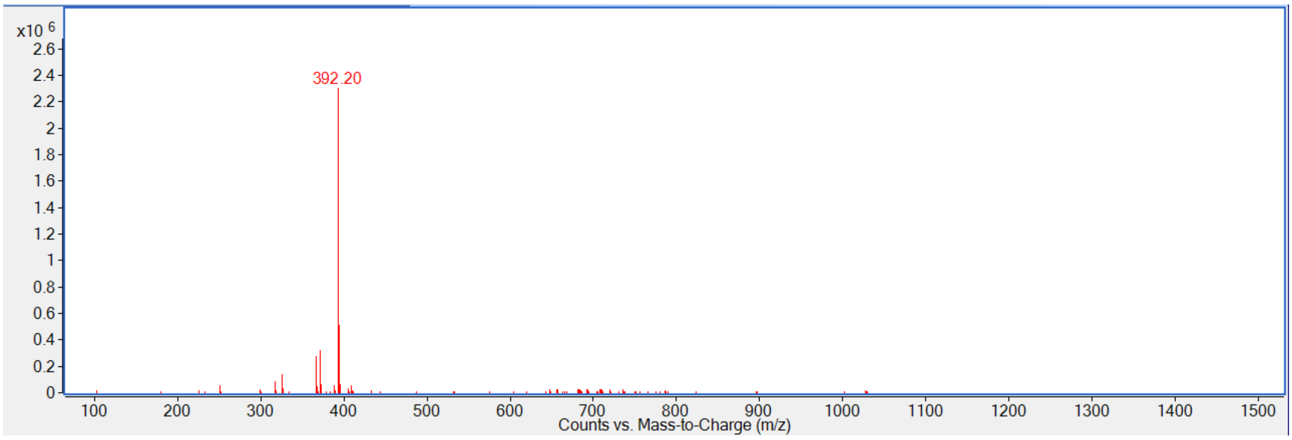

Figure S3. ESI-MS spectrum of metacycloprodigiosin (**1**), recorded in positive mode.

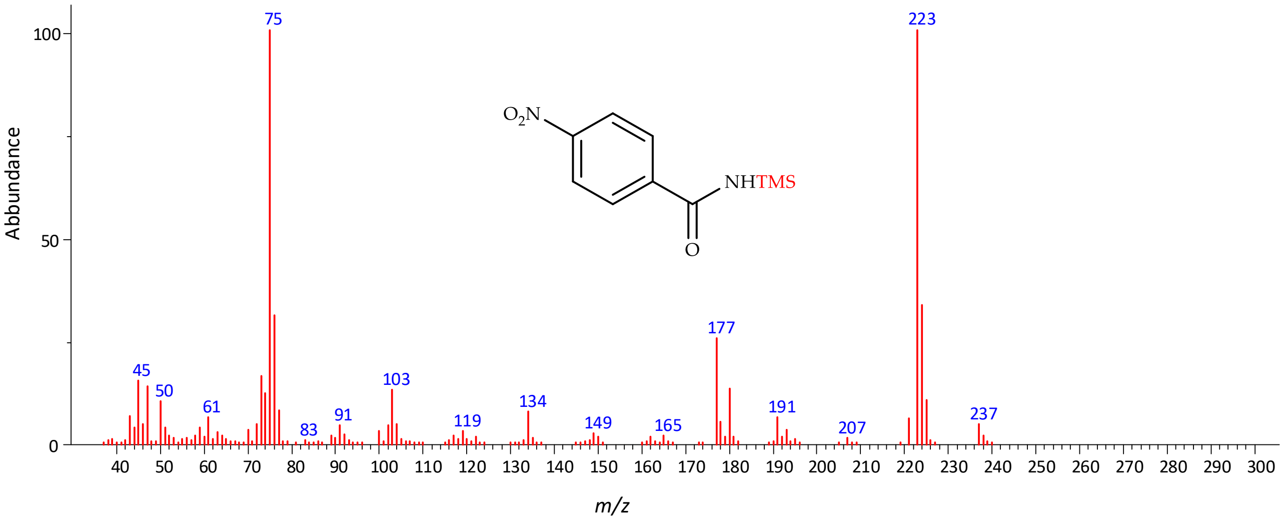

Figure S4. EI mass spectrum at 70 eV of 4-nitrobenzamide, TMS (RI = 1864). TMS = trimethylsilyl group.

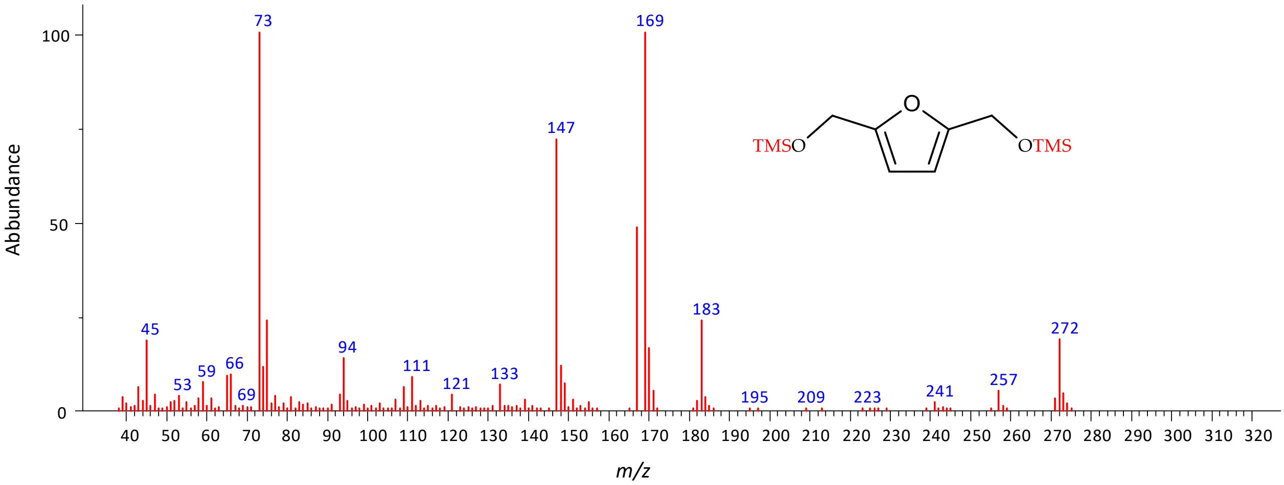

Figure S5. EI mass spectrum at 70 eV of furandimethanol, 2TMS (RI = 1427). TMS = trimethylsilyl group.

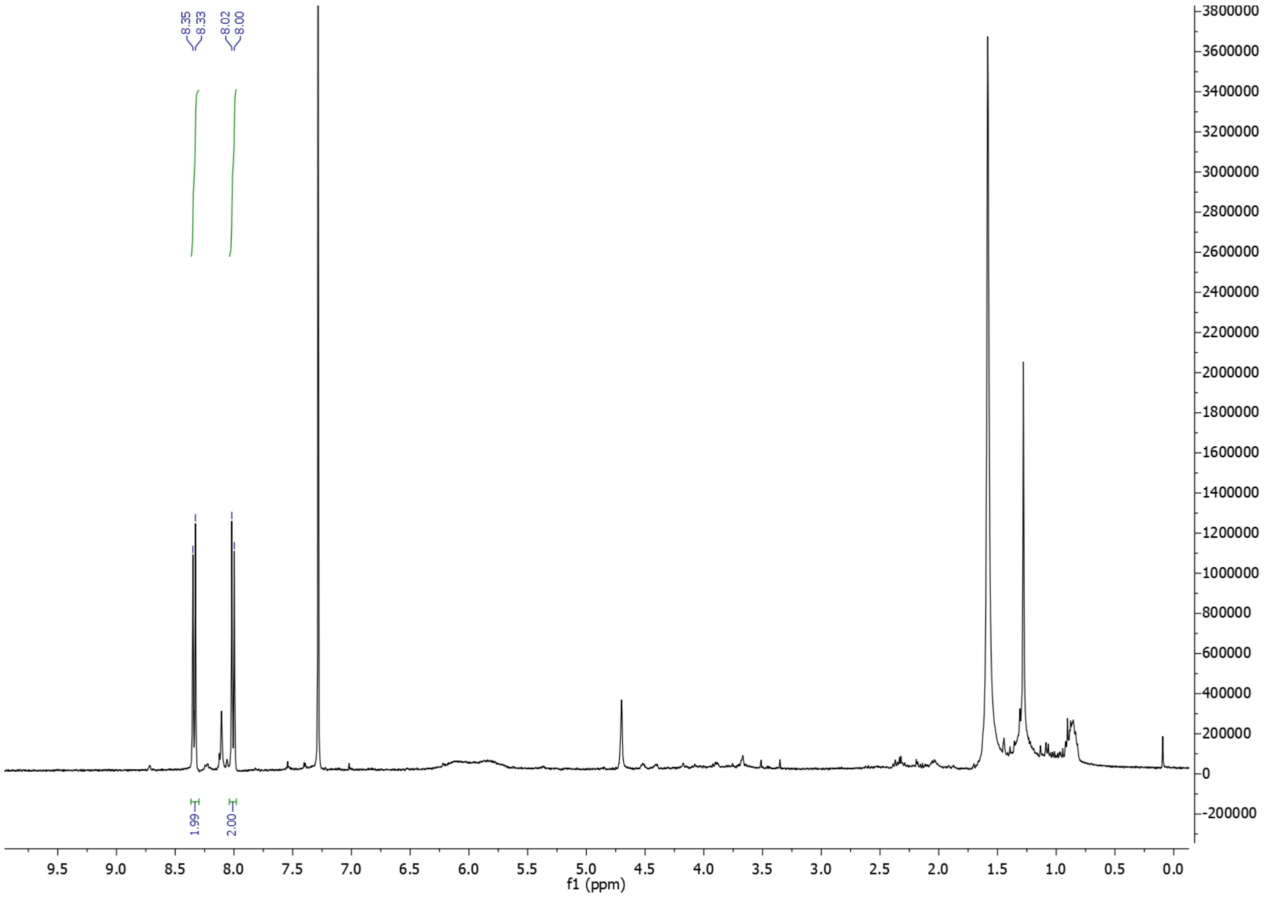

Figure S6. ^{1}H NMR spectrum of FC ($CHCl_3$, 400 MHz).

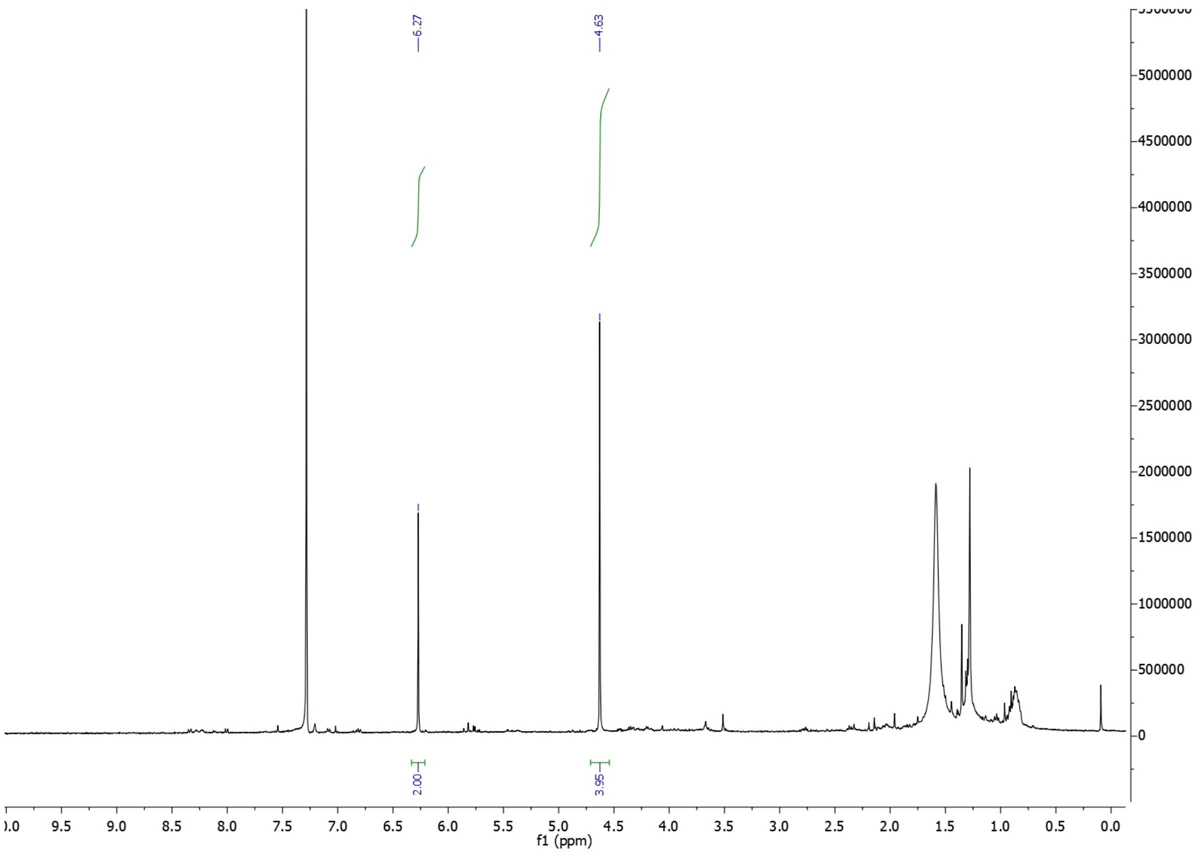

Figure S7. ^{1}H NMR spectrum of FE ($CHCl_3$, 400 MHz).
